# Association between improved metabolic risk factors and perceived fatigue during dietary intervention trial in relapsing-remitting multiple sclerosis: A secondary analysis of the WAVES trial

**DOI:** 10.3389/fneur.2022.1022728

**Published:** 2023-01-19

**Authors:** Aneli T. Villa, Betty H. Tu, Tyler J. Titcomb, Solange M. Saxby, Farnoosh Shemirani, Patrick Ten Eyck, Linda M. Rubenstein, Linda G. Snetselaar, Terry L. Wahls

**Affiliations:** ^1^Department of Internal Medicine, University of Iowa, Iowa City, IA, United States; ^2^Department of Epidemiology, University of Iowa, Iowa City, IA, United States; ^3^Institute for Clinical and Translational Science, University of Iowa, Iowa City, IA, United States

**Keywords:** multiple sclerosis, fatigue, low-saturated fat diet, modified Paleolithic elimination diet, cholesterol, weight, insulin

## Abstract

**Background:**

Preliminary dietary intervention trials with the low-saturated fat (Swank) and modified Paleolithic elimination (Wahls) diets have shown favorable effects on fatigue among people with multiple sclerosis (MS); however, their impact on metabolic health is unknown.

**Objective:**

To evaluate the impact of the Swank and Wahls diets on markers of metabolic health and to determine the association and mediation effect between changes in metabolic health and perceived fatigue among people with relapsing-remitting MS (RRMS).

**Methods:**

As part of a randomized parallel-arm trial, vital signs, blood metabolic biomarkers, and the fatigue scale for motor and cognitive functions (FSMC) were collected from participants with relapsing-remitting MS (*n* = 77) at four study visits spaced 12 weeks apart: (1) run-in, (2) baseline, (3) 12-weeks, and (4) 24-weeks. Participants followed their usual diet at run-in, then were randomized at baseline to either the Swank or Wahls diets and followed for 24 weeks.

**Results:**

Both groups had significant reductions in weight, body mass index (BMI), total cholesterol, and low-density lipoprotein (LDL) at 12- and 24-weeks compared to respective baseline values (*p* ≤ 0.04 for all). The Swank group also had a significant reduction in high-density lipoprotein (HDL) at 12- and 24-weeks (*p* = 0.0001 and *p* = 0.02, respectively), while the Wahls group had significant reductions in diastolic blood pressure (DBP). In addition, both groups had significant reductions in FSMC total perceived fatigue and the motor and cognitive fatigue subscales at 12- and 24-weeks (*p* ≤ 0.01 for all); however, change in the cognitive subscale was not significant at 12-weeks in the Swank group (*p* = 0.06). Furthermore, the favorable effects, of both diets, on markers of metabolic health were not associated with and did not mediate the effect of the diets on perceived fatigue (*p* > 0.05 for all).

**Conclusion:**

Both diets lead to significant reductions in perceived fatigue, weight, BMI, total cholesterol, and LDL, but the significant reductions in perceived fatigue were independent of changes in markers of metabolic health.

## 1. Introduction

Multiple sclerosis (MS) is a chronic, immune-mediated, demyelinating disease of the central nervous system that is increasing in prevalence in the United States (1). Disease progression leads to increased perceived fatigue (2), which is among the most common and disabling symptoms of MS (3). As such, many people seek non-pharmacologic approaches like dietary changes to relieve MS-related symptoms (4).

Studies have shown that people with MS have great interest in alternative approaches, including diet, to manage symptoms and improve wellness (5). Two popular diets in the MS community are the low-saturated fat diet developed by Dr. Roy Swank and the modified Paleolithic diet developed by Dr. Terry Wahls (6, 7). Both the Swank and Wahls diets were shown to significantly reduce fatigue and improve quality of life in people with relapsing-remitting MS (RRMS) (8). Similarly, a recent systematic review and network meta-analysis found that several food-based diets including the Paleolithic, Mediterranean, and low-fat (Swank and McDougall) diets, which are high in fruits and vegetables and low in ultra-processed foods, reduce perceived fatigue and improve physical and mental quality of life among people with MS (9). However, due to the small sample sizes, high risk of bias, and methodological issues among the preliminary studies, evidence is insufficient to support any specific therapeutic diet for MS (9–11), and the mechanism by which dietary changes lead to these improvements remains elusive (12).

One possible mechanism by which diet may lead to favorable outcomes is by improving metabolic health. Studies have shown that metabolic risk factors, such as obesity, hyperlipidemia, and hypertension, are common among people with MS (13). These metabolic risk factors are dependent on diet (14) and are associated with increased relapses, lesion burden, and lower brain volumes (15, 16). Obesity among people with MS is associated with greater fatigue, increased relapses, higher disability, and lower quality of life (17, 18). Furthermore, people with MS have an increased risk of ambulatory disability and lower brain volume if they have one or more vascular comorbidities such as hyperlipidemia or hypertension (19, 20).

Furthering the understanding of the impact of dietary modifications on metabolic health may elucidate the mechanisms by which diet improves MS-related fatigue and lead to new adjunct treatment options. Previous studies investigating the modified Paleolithic diet have shown improvements in lipid profiles but not in glucose or insulin (21, 22). Both the Swank and Wahls diets are associated with clinically significant reductions in perceived fatigue and quality of life in people with RRMS (8); however, their impact on metabolic health is unclear. The goal of this secondary analysis is to evaluate the effects of these diets on biomarkers of metabolic health [weight, BMI, blood pressure, glucose, insulin, cholesterol, high-density lipoprotein (HDL), low-density lipoprotein (LDL), triglycerides, and hemoglobin A1c] and determine the relationship between diet-induced changes in metabolic risk factors and perceived fatigue among individuals with RRMS.

## 2. Methods

### 2.1. Participants and study design

This is a secondary analysis of a 36-week, randomized, parallel-group, single-blinded trial conducted at the University of Iowa Prevention Intervention Center that showed that both the Swank and Wahls diets cause significant improvements in quality of life and reductions in fatigue (8). The University of Iowa Institutional Review Board approved the trial and followed the Consolidated Standards of Reporting Trials (CONSORT) reporting guidelines (23). Written and informed consent was obtained from all study participants.

Adult participants between 18 and 70 years of age were recruited from Iowa City, Iowa, and the surrounding 500-mile area. Participants were eligible for enrollment in the study if they: (1) had neurologist-confirmed RRMS consistent with the 2010 McDonald Criteria (24), (2) had moderate to severe fatigue, (3) possessed the ability to walk 25 feet with unilateral support, (4) were not pregnant nor planning to become pregnant, and (5) were willing to comply with all study procedures. Major exclusion criteria included: (1) MS-relapse or change in disease-modifying drug therapy within the 12 weeks prior to the start of the study; (2) any change in medication for management of MS-related symptoms within 12 weeks prior to the start of the study; (3) BMI <19 kg/m^2^; (4) severe mental impairment (i.e., schizophrenia); (5) self-reported adverse reactions to gluten-containing foods; (6) diagnosed with comorbidities including celiac disease, severe psychiatric disorders, eating disorders, kidney stones, heart failure, angina, or cirrhosis; (7) taking insulin or warfarin; and (8) undergoing radiation or chemotherapy. Complete inclusion and exclusion criteria are listed in the trial protocol (25).

### 2.2. Study procedures

Following a 12-week observational run-in phase, participants were randomized 1:1 at baseline to either the low-saturated fat (Swank) diet or the modified Paleolithic elimination (Wahls) diet. Study diet education was provided by intervention registered dietitians (RDs) at baseline. The RDs provided active diet support consisting of in-person and telephone-based nutrition counseling sessions for the first 12 weeks of the intervention. During the second half of the intervention period, the active RD support was discontinued, but participants could still contact the RDs anytime for support or assistance. Biospecimen and data collection occurred during four study visits, each spaced 12 weeks apart: (1) run-in, (2) baseline, (3) 12-weeks, and (4) 24-weeks.

### 2.3. Study diets

Participants in both the Swank and Wahls diet groups were instructed to follow their assigned diet *ad libitum* for the 24-week intervention period after randomization. The composition of both diets has been reviewed in detail elsewhere (7). Briefly, the Swank diet limits saturated fat to ≤15 g per day and provides 20–50 g of unsaturated fat, four servings of grains, and four servings of fruits and vegetables (FV) per day. The Wahls diet recommends 6–9 servings of FV and 6–12 ounces of meat per day, depending on gender. All grains, legumes, eggs, and dairy (except clarified butter and ghee) are excluded from the Wahls diet. Nightshade vegetables were also excluded in this group between the baseline and the 12-week timepoint; then, participants were guided to reintroduce nightshades between the 12-week and 24-week time points. Additionally, all study participants followed the same daily supplement regimen (25). Participants were emailed personalized feedback on their diet checklists every 4 weeks to encourage study diet adherence, which was previously reported as 80% for the Wahls group and 87% for the Swank group based on analysis of 3-day weighed food records at 12 weeks (8).

### 2.4. Outcomes

The primary outcomes, dietary characteristics, and qualitative interviews from this trial have been published previously (8, 26, 27). For this secondary analysis, perceived fatigue was evaluated with the Fatigue Scale for Motor and Cognitive Functions (FSMC). The FSMC is a reliable and validated 20-item questionnaire that assesses total as well as the motor and cognitive domains of fatigue in people with MS (28). For the total fatigue FSMC score, values range from 20 to 100, where higher scores indicate more severe fatigue. Clinical outcomes, including height, weight, and blood pressure, were collected using standardized procedures by trained staff. Blood biospecimens were collected by phlebotomists and sent to the Iowa City Veteran's Affairs Department of Pathology for analysis of metabolic biomarkers, including glucose, hemoglobin A1c, insulin, total cholesterol, low-density lipoprotein (LDL), and high-density lipoprotein (HDL). Clinically important differences were defined for this study as follows: 5% change for weight and BMI; 10% change for total cholesterol, LDL, HDL, glucose, and insulin; 5 mm Hg change for systolic and diastolic blood pressure; 30% change for triglycerides; 0.5% change for hemoglobin A1c (29); and half the difference between severe and moderate fatigue cutoffs (i.e. 5 points for total fatigue and 2.5 points for the motor and cognitive fatigue subscales) (28).

### 2.5. Statistical analysis

Descriptive statistics were calculated for every variable at enrollment using frequencies, means ± standard error of the mean (SEM), and medians (interquartile range). Outliers were checked for accuracy and possible data entry errors. Distributions of continuous variables were evaluated for normality by graphical observation. Data from all participants completing 12- and 24-week assessments were included in intention-to-treat analyses.

Generalized linear mixed models (30) were used to test the interacting effects of diet and time on outcome measures while accounting for repeated measures for each participant. The identity link function was used for normally distributed outcomes, while the log link function was used for right-skewed continuous (gamma distribution) and count (Poisson distribution) measures. Other potentially important variables (age, sex, BMI, smoking status, alcohol consumption, walking assistance, years since MS diagnosis, disease-modifying drug therapy, baseline vitamin D, baseline 6-min walk distance) were considered for inclusion in each model to assess their relationship with the outcome and whether they modified the estimates for the diet and time interaction. For each outcome, the model with the smallest Akaike information criterion (AIC) (31) was deemed to have the optimal predictor set. Point estimates, 95% confidence intervals, and *p*-values of the within- and between-group mean changes in outcome measures over study visits were generated for each optimal model. In addition, a secondary per-protocol analysis was conducted excluding participants who did not adhere to their assigned diet as defined by ≤0.2 g gluten for the Wahls group (20%) and ≤18 g saturated fat for the Swank group (13%) (8).

To evaluate the association of 12-week changes in metabolic risk factors and fatigue, linear mixed models controlling for diet and baseline values were conducted. Slope estimates, 95% confidence intervals, and *p*-values for the association with fatigue were generated for each metabolic risk factor. Causal mediation analyses were used to determine the mediation effect for the interaction between metabolic risk factor values and time on fatigue from baseline to 12 weeks controlling for diet assignment. All analyses were conducted as two-sided tests (α = 0.05) using SAS software (version 9.4, SAS Institute, Inc.).

## 3. Results

**Figure 1:**
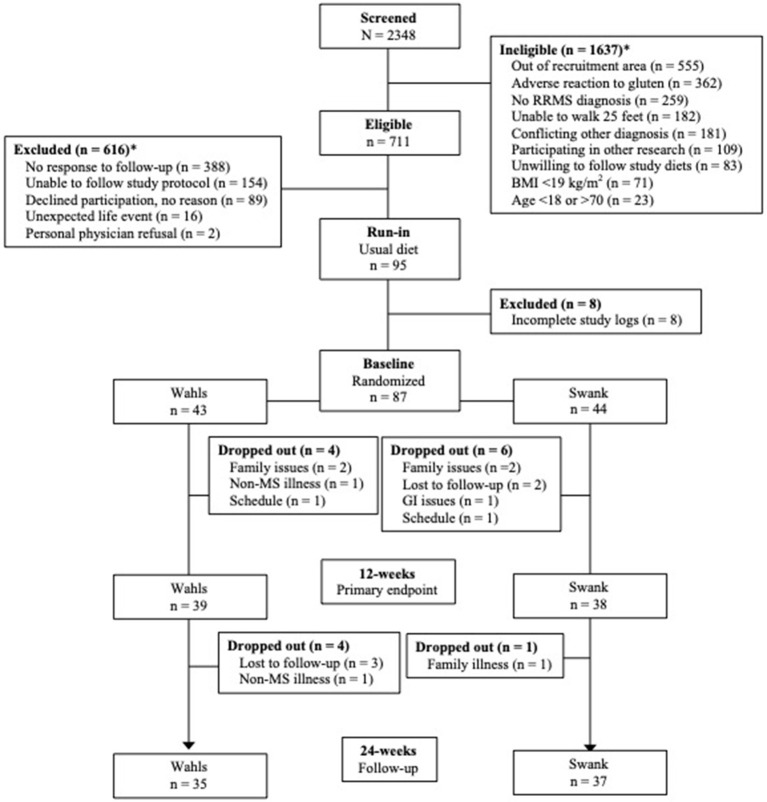
CONSORT diagram of study recruitment and participant flow. *Reasons for ineligibility or exclusion may not add up to the total of ineligible or excluded because some participants were found ineligible or were excluded for multiple reasons. Adapted from Wahls et al. (8).

This secondary analysis included 77 participants (39 Wahls and 38 Swank) who completed the primary study endpoint at 12-weeks and 72 participants (35 Wahls and 37 Swank) completed follow-up to 24 weeks (Figure 1). At baseline, there were no significant between-group differences (Table 1), and baseline metabolic risk factor values were consistent with pre-randomization run-in values (Table 2). Participants in the Swank group reduced their intake of energy by 201 ± 86.0 kcals (*p* = 0.02) and the Wahls group reduced their intake of energy by 447 ± 84.8 kcals (*p* < 0.001) compared to their respective baseline intake (*p* = 0.003 between groups).

**Table 1:** Baseline characteristics of participants with RRMS who completed the primary study endpoint at 12-weeks.

**Characteristics**	**Swank**	**Wahls**	***p*-value^a^**
**N**	38	39	
**Age (years)**	46.9 ± 1.7	46.4 ± 1.5	0.84
**Gender (female)**	35 (92.1)	32 (82.1)	0.31
**MS duration (years)**	12.1 ± 1.6	9.3 ± 1.0	0.14
**Disease modifying drug use**			0.83
None	13	10	
Oral	11	11	
Injectable	10	12	
Infused	4	6	
**Race (Caucasian)**	36 (94.7)	38 (97.4)	0.99
**Education**			0.32
High school	0 (0.0)	3 (7.7)	
Some college	12 (31.6)	10 (25.6)	
4-year degree	11 (28.9)	8 (20.5)	
Advanced degree	15 (39.5)	18 (46.2)	
**Smoking status**			0.13
Never	29 (76.3)	23 (59.0)	
Former	3 (7.9)	2 (5.1)	
Current	6 (15.8)	14 (35.9)	
**Alcohol drinks per month^b^**			0.99
None	6 (15.8)	7 (17.9)	
Within recommendations	29 (76.3)	29 (74.4)	
Above recommendations	3 (7.9)	3 (7.7)	
**FSMC total fatigue^c^**	45.2 ± 2.24	47.4 ± 2.17	0.47
Cognitive fatigue	20.4 ± 1.24	22.7 ± 1.23	0.19
Motor fatigue	24.7 ± 1.39	24.7 ± 1.18	0.98

All values are mean ± SEM or N (%). Adapted from Wahls et al. (8).

a Significance determined by Fisher's exact test or generalized linear models.

b Alcohol recommendations were defined as ≤1 or ≤2 standard drinks for females and males respectively.

c Participants were randomized based on baseline fatigue scores to ensure balance between groups.

**Table 2:** Metabolic risk factor values among participants with RRMS assigned to the Swank or Wahls dietary interventions.

**Biomarker**	**Study visit**			
	**Run-in**	**Baseline**	**12 weeks**	**24 weeks**
**Swank**				
Systolic BP (mmHg)	114 ± 2.36	115 ± 2.43	113 ± 2.65	115 ± 1.87
Diastolic BP (mmHg)	74.0 ± 1.64	73.4 ± 1.67	72.7 ± 1.68	72.2 ± 1.84
Weight (kg)	77.1 ± 2.92	77.3 ± 2.92	75.4 ± 3.11	75.3 ± 3.35^*^
BMI (kg/m^2^)	27.6 ± 0.92	27.6 ± 0.92	26.8 ± 0.94^***^	26.7 ± 1.03^***^
Glucose (mg/dl)	92.4 ± 1.23	92.2 ± 1.55	90.5 ± 1.51	91.6 ± 1.41
A1c (%)	5.31 ± 0.06	5.27 ± 0.06	5.29 ± 0.07	5.21 ± 0.06
Insulin (μIU/ml)	6.17 ± 0.76	6.52 ± 0.83	6.10 ± 0.98	6.12 ± 1.03
Cholesterol (mg/dl)	190 ± 5.62	189 ± 5.70	170 ± 4.35^***^^,^^†^	174 ± 5.23^***^
HDL (mg/dl)	65.5 ± 2.77	63.0 ± 2.28	57.3 ± 2.05^***^	59.6 ± 2.25^*^
LDL (mg/dl)	122 ± 5.09	123 ± 5.25	108 ± 4.39^***^	109 ± 4.92^***^
Triglycerides (mg/dl)	91.2 ± 7.35	92.7 ± 8.65	85.3 ± 7.05	82.4 ± 6.44
**Wahls**				
Systolic BP (mmHg)	116 ± 1.87	117 ± 2.38	115 ± 2.11	116 ± 2.45
Diastolic BP (mmHg)	76.5 ± 1.53	77.3 ± 1.72	73.2 ± 1.53^**^	73.4 ± 1.86^**^
Weight (kg)	85.0 ± 3.28	85.3 ± 3.30	80.3 ± 3.04^***^	78.0 ± 2.74^**^
BMI (kg/m^2^)	30.1 ± 1.24	30.2 ± 1.25	28.4 ± 1.18^***^	28.4 ± 1.32^***^
Glucose (mg/dl)	101 ± 5.27	98.9 ± 3.94	97.0 ± 3.36	98.5 ± 4.43
A1c (%)	5.56 ± 0.21	5.47 ± 0.16	5.34 ± 0.11	5.38 ± 0.12
Insulin (μIU/ml)	7.12 ± 1.13	8.84 ± 1.56	4.84 ± 0.47^**^	5.05 ± 0.63^*^
Cholesterol (mg/dl)	198 ± 6.84	198 ± 7.28	184 ± 6.04^**^^,^^†^	187 ± 6.32^*^
HDL (mg/dl)	65.4 ± 2.74	65.5 ± 2.71	63.7 ± 2.84	65.9 ± 3.00
LDL (mg/dl)	127 ± 5.93	127 ± 6.24	114 ± 5.06^**^	114 ± 5.91^***^
Triglycerides (mg/dl)	111 ± 8.59	105 ± 8.02	86.6 ± 7.68^**^	79.9 ± 5.97^***^

All values are mean ± SEM.

Within-group statistical significance compared to baseline values indicated by

* p ≤ 0.05,

** p ≤ 0.01, and

*** p ≤ 0.001.

Between-group statistically significant differences indicated by

† p ≤ 0.05.

**Figure 2:**
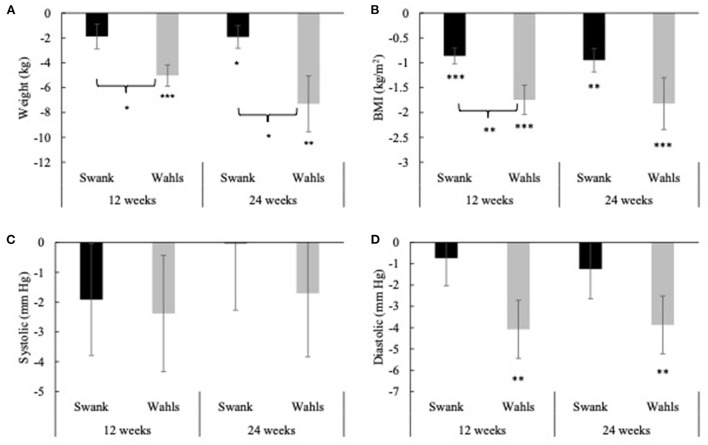
Mean change from baseline for **(A)** weight, **(B)** BMI, **(C)** systolic blood pressure, and **(D)** diastolic blood pressure at 12- and 24-weeks for the Swank (black bars) and Wahls (gray bars) groups. Statistical significance was determined by generalized linear mixed models and represented by *p ≤ 0.05, **p ≤ 0.01, and ***p ≤ 0.001.

Clinically and statistically significant mean (±SEM) reductions in weight were observed at both 12- (−5.02 ± 0.86 kg, *p* < 0.0001) and 24-weeks (−7.31 ± 2.25 kg, *p* = 0.002) in the Wahls group, while in the Swank group statistically significant weight loss occurred only at 24 weeks (−1.92 ± 0.91 kg, *p* = 0.04; Figure 2A). Similarly, statistically significant mean (±SEM) reductions in BMI were observed at 12- and 24-weeks for both the Wahls (−1.75 ± 0.30 kg/m^2^ and −1.82 ± 0.52 kg/m^2^, respectively; *p* < 0.001 for both) and the Swank (−0.86 ± 0.16 and −0.95 ± 0.24 kg/m^2^, respectively; *p* < 0.0001 for both) groups (Figure 2B) although only the Wahls group experienced clinically significant reductions in BMI. The Wahls group had lost significantly more weight than the Swank group at both 12 weeks (−3.14 ± 1.32 kg, *p* = 0.02) and 24 weeks (−5.38 ± 2.42 kg, *p* = 0.03). Similarly, the Wahls group had a significantly greater decrease in BMI compared to the Swank diet at 12 weeks (−0.89 ± 0.34 kg/m^2^, *p* = 0.009). There were no significant changes in systolic blood pressure (SBP) within or between groups (Figure 2C). Statistically significant mean (±SEM) reductions of −4.08 ± 1.36 (*p* = 0.003) and −3.87 ± 1.36 (*p* = 0.005) in diastolic blood pressure (DBP) were observed in the Wahls group at 12- and 24-weeks, respectively (Figure 2D), while the Swank group did not change from baseline values. There were no differences between groups in DBP.

**Figure 3:**
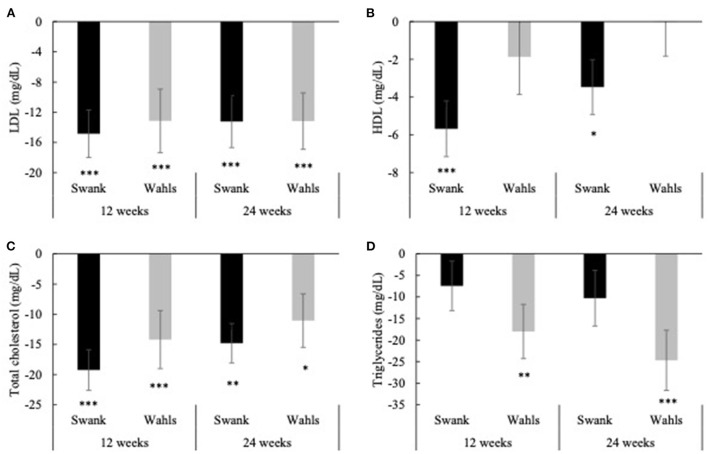
Mean change from baseline for **(A)** LDL, **(B)** HDL, **(C)** total cholesterol, and **(D)** triglycerides at 12- and 24-weeks for the Swank (black bars) and Wahls (gray bars) groups. Statistical significance was determined by generalized linear mixed models and represented by *p ≤ 0.05, **p ≤ 0.01, and ***p ≤ 0.001.

Both groups had clinically and statistically significant reductions in LDL cholesterol at 12 and 24-weeks compared to baseline (Figure 3A). The Swank group had mean (±SEM) reductions of −14.85 ± 3.14 and −13.24 ± 3.45 (*p* < 0.0001 and *p* = 0.0001) mg/dl at 12 and 24-weeks and the Wahls group had mean (±SEM) reductions of −13.15 ± 4.20 and −13.18 ± 3.74 (*p* = 0.002 and *p* = 0.0004) mg/dl at 12 and 24 weeks, respectively. The Swank group had statistically significant mean (±SEM) reductions in HDL cholesterol at 12- and 24-weeks from baseline values with mean differences of −5.68 ± 1.48 (*p* = 0.0001) and −3.47 ± 1.45 (*p* = 0.02) mg/dl, respectively, while the Wahls group did not change (Figure 3B). Both groups had statistically significant within-group reductions in total cholesterol compared to baseline values. The Swank group had mean (±SEM) reductions of −19.24 ± 3.36 and −14.80 ± 3.26 mg/dl at 12- and 24-weeks, respectively (*p* < 0.0001 for both), and the Wahls group had mean (±SEM) reductions of −14.20 ± 4.80 (*p* = 0.003) and −11.06 ± 4.43 (*p* = 0.02) mg/dl at 12- and 24-weeks, respectively (Figure 3C). The Wahls group had statistically significant mean (±SEM) reductions in triglycerides at the 12- and 24-weeks with mean (±SEM) differences from baseline of −18.01 ± 6.30 (*p* = 0.004) and −24.71 ± 6.98 (*p* = 0.0004) mg/dl, respectively, whereas the Swank group did not change from baseline values (Figure 3D). There were no significant between-group differences in mean changes from baseline for triglycerides, total, LDL, or HDL cholesterol.

**Figure 4:**
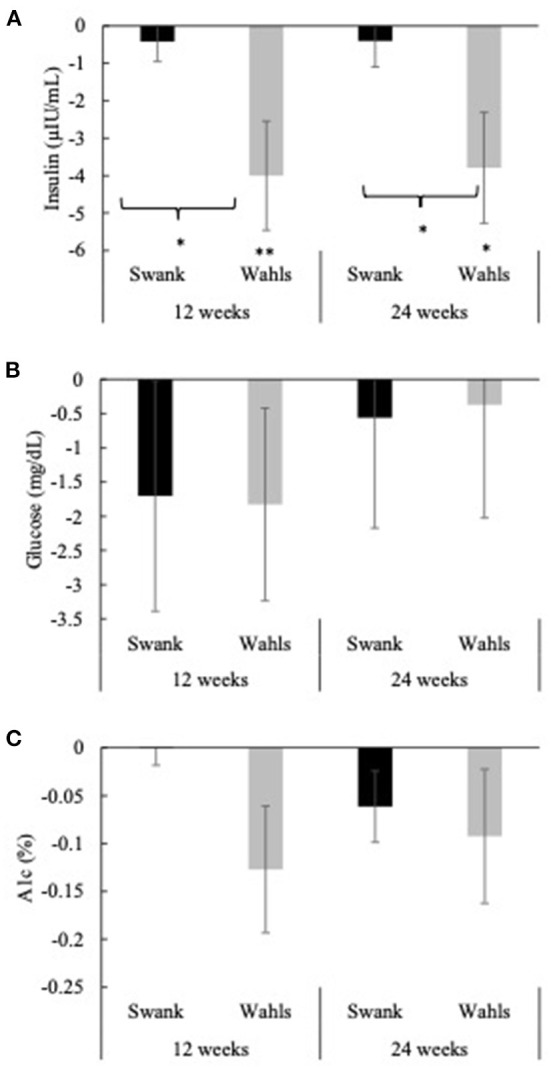
Mean change from baseline for **(A)** insulin, **(B)** glucose, and **(C)** hemoglobin A1c at 12- and 24-weeks for the Swank (black bars) and Wahls (gray bars) groups. Statistical significance was determined by generalized linear mixed models and represented by *p ≤ 0.05 and **p ≤ 0.01.

The Wahls group had clinically and statistically significant mean (±SEM) reductions in insulin at 12- and 24-weeks with mean differences from baseline of −4.00 ± 1.46 (*p* = 0.006) and −3.79 ± 1.48 (*p* = 0.02) μIU/mL, respectively, whereas insulin values among the Swank group did not change from baseline values (Figure 4A). The Wahls group had significantly greater reductions in insulin compared to the Swank group at 12- and 24-weeks (*p* < 0.05 for both). There were no significant within- or between-group differences observed for glucose or hemoglobin A1c (Figures 4B, C). All significant changes in metabolic risk factors were maintained or strengthened in the per-protocol analysis excluding participants who were not adherent to their respective diet assignments at 12-weeks (Supplementary Table 1).

**Figure 5:**
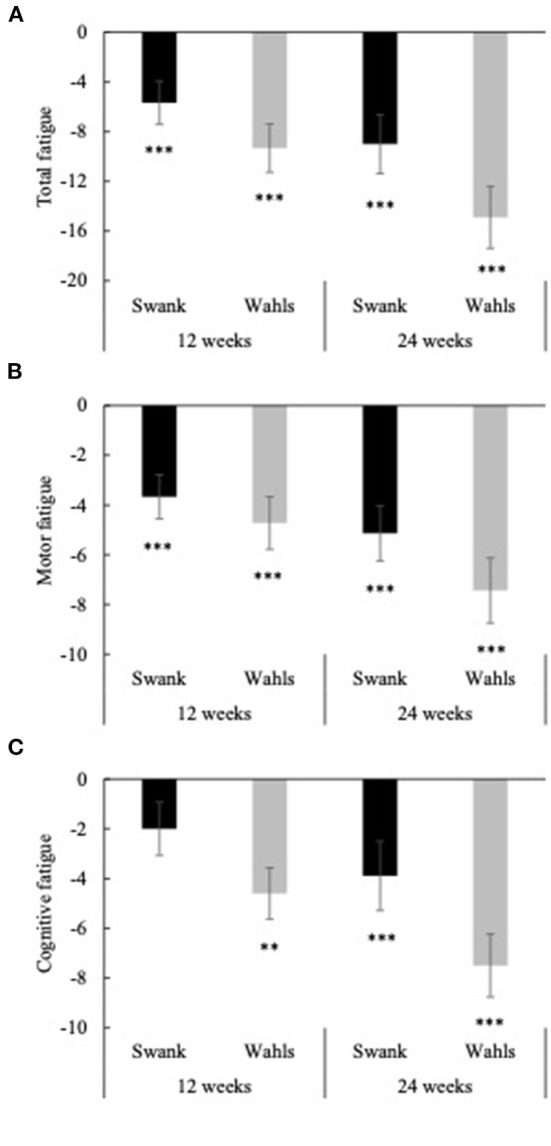
Mean change from baseline for **(A)** total fatigue, **(B)** cognitive fatigue, and **(C)** motor fatigue as determined by the fatigue scale for motor and cognitive functions (FSMC) at 12- and 24-weeks for the Swank (black bars) and Wahls (gray bars) groups. Statistical significance was determined by generalized linear mixed models and represented by *p ≤ 0.05 and ***p ≤ 0.001.

Clinically and statistically significant mean (±SEM) reductions from baseline were observed in total perceived fatigue (FSMC) at both 12 and 24 weeks for the Swank (−5.69 ± 1.73 and −9.00 ± 2.37, respectively; *p* ≤ 0.001 for both) and Wahls (−9.33 ± 1.96 and −14.9 ± 2.48, respectively; *p* ≤ 0.0001 for both) groups (Figure 5A). Similar clinically and statistically significant mean (±SEM) reductions were observed in the FSMC motor and cognitive subscales among both groups at 12 and 24 weeks (*p* ≤ 0.01 for all; Figures 5B, C); however, among the Swank group, the change in the cognitive subscale was not significant at 12 weeks (*p* = 0.06). All significant within-group changes in fatigue were maintained or strengthened in the per-protocol analysis excluding participants who were not adherent to their respective diet assignment at 12-weeks; however, the Wahls group had significantly greater reductions in total and cognitive fatigue compared to the Swank group at 24-weeks in the sensitivity analysis (Supplementary Figure 1). The decreases in FSMC scores were not associated with or mediated by changes in metabolic risk factors (Table 3).

**Table 3:** Association and mediation effect of 12-week metabolic risk factor changes on perceived fatigue changes among participants with RRMS enrolled in a dietary intervention study.

**Risk factor (unit)**	**FSMC total**			
	**β-coefficient (95% CI)**	***p***	**Percentage mediated (95% CI)**	***p***
Systolic BP (mmHg)	−0.17 (−0.42, 0.09)	0.20	−5.78 (−21.4, 9.82)	0.47
Diastolic BP (mmHg)	−0.31 (−0.69, 0.06)	0.10	−10.5 (−29.0, 7.92)	0.26
Weight (kg)	0.33 (−0.31, 0.98)	0.31	2.63 (−7.14, 12.4)	0.60
BMI (kg/m^2^)	0.91 (−0.93, 2.75)	0.33	0.92 (−8.81, 10.7)	0.85
Glucose (10 mg/dl)	0.78 (−2.59, 4.15)	0.65	−0.97 (−6.39, 4.45)	0.73
A1c (%)	−1.25 (−13.5, 11.0)	0.84	−1.54 (−9.71, 6.64)	0.71
Insulin (μIU/ml)	−0.30 (−1.01, 0.41)	0.41	6.06 (−14.9, 27.0)	0.57
Cholesterol (10 mg/dl)	−0.43 (−1.69, 0.84)	0.50	8.86 (−14.9, 32.6)	0.46
HDL (10 mg/dl)	−0.57 (−3.40, 2.25)	0.69	−1.01 (−11.0, 9.02)	0.84
LDL (10 mg/dl)	−0.13 (−1.56, 1.30)	0.86	11.8 (−12.5, 36.0)	0.34
Triglycerides (10 mg/dl)	−0.50 (−1.35, 0.34)	0.24	−2.29 (−14.9, 10.3)	0.72

All values shown as β-coefficient or percentages and 95% confidence intervals.

## 4. Discussion

In this secondary analysis of the WAVES trial, both the Swank and Wahls diets were shown to significantly reduce weight, BMI, total cholesterol, and LDL; however, these improvements in metabolic risk factors were not associated with and did not mediate the significant reductions in perceived fatigue among adults with RRMS.

Both diets significantly improved total cholesterol and LDL levels, and the Wahls diet was also associated with improved triglyceride levels. Prior evidence suggests that lipid profiles are inversely associated with fatigue in people with MS (21, 32, 33); however, the present study did not observe a relationship between lipid biomarkers and fatigue. In addition, poor lipid profiles, including high total cholesterol, LDL, and triglyceride levels, have also been linked with worse EDSS, increased disability, greater brain atrophy, and increased presence of contrast-enhancing lesions on brain MRI scans (33). Interestingly, HDL levels remained stable in the Wahls group, while the Swank group experienced statistically significant reductions in HDL levels over the duration of this study. Given the direct link between dietary saturated fat and HDL levels (34), it is not surprising that HDL was lowered in the Swank group; however, it is concerning as prior research has observed an association between higher HDL levels and lower contrast-enhancing lesion volume in people with MS (33).

Both the Swank and Wahls diets promoted significant weight loss and reduced BMI, which is unsurprising as both *ad libitum* dietary interventions lead to decreased intake of energy and recommend a high intake of fruits, vegetables, and unsaturated fats and limit intake of ultra-processed foods (7). Prior research has shown that people with MS, who are also overweight or obese, are more likely to have additional comorbidities, which in turn are associated with increased odds of disability and MS relapse (18). In addition, evidence suggests that hypertension is associated with more advanced white matter and whole brain atrophy, greater physical disability, and slower walking speeds among people with MS (35, 36). Diastolic blood pressure in the Swank group remained stable over the duration of the study, in contrast to the Wahls group, which experienced statistically significant reductions in DBP. No significant changes in SBP were observed in this study, nor were changes in SBP or DBP associated with changes in perceived fatigue.

Neither group had significant changes in glucose or hemoglobin A1c levels, though the Wahls diet did have clinically and statistically lower insulin levels at 12 and 24 weeks; however, changes in insulin, glucose, and hemoglobin A1c levels were not associated with changes in perceived fatigue. These results are consistent with prior research, which showed that a Paleolithic diet leads to significantly lower plasma insulin and improves insulin sensitivity among obese individuals without MS (37). In addition, the present findings confirm those from a preliminary study among people with MS that observed small reductions in fasting insulin levels that were not significant, due, in part, to small sample size, after a 12-week intervention with a modified Paleolithic diet (22). The reduction in insulin levels observed among the Wahls group in this study may be due to the recommendation for lower intake of energy or carbohydrate-containing foods compared to the Swank diet (7).

The strengths of this study include robust analytical methods, large sample size, and objective measures of the metabolic risk factors. Additionally, there was high diet adherence (≥80%) among both groups as evidenced by analysis of weighed food records, and the present secondary fatigue outcomes are consistent with the primary outcomes previously reported (8). This study is limited by the lack of diversity of study participants, lack of the usual diet comparison group, short intervention duration, lack of evaluation of disease activity, and the wide range of exclusion criteria which limits the generalizability of the findings to fatigued people with RRMS. In addition, it should be noted that while no statistically significant differences existed between the groups at baseline, the Wahls group had higher baseline values for all metabolic risk factors measured and had a significantly lower *ad libitum* intake of energy. Therefore, although there were statistically significant differences between the Swank and Wahls groups in terms of the magnitude of change in weight, BMI, and insulin levels, these differences between the two groups make it difficult to interpret the between group findings.

People with MS frequently report making dietary changes (4). The results from this study suggest that diet-induced improvement in perceived fatigue among people with RRMS is independent of improvements in metabolic health. Despite the limitations of the present study, this secondary analysis provides compelling rationale for future randomized controlled trials with longer duration, larger sample size, and brain MRI-evaluated disease activity to explore the intersection between dietary interventions and the MS disease course.

## Author's note

Aspects of this work were presented at CMSC 2021 and ACTRIMS Forum 2022.

## Data availability statement

The raw data supporting the conclusions of this article will be made available by the authors, without undue reservation upon request.

## Ethics statement

The studies involving human participants were reviewed and approved by University of Iowa Institutional Review Board. The patients/participants provided their written informed consent to participate in this study.

## Author contributions

AV and BT wrote the first draft of the manuscript. TT cleaned the data. TT, FS, and PT performed all statistical analyses. LR managed unblinded data and confirmed statistical analyses. SS and FS revised the manuscript. TW and LS designed the study and oversaw all study procedures. All authors have read and approved the final version of the manuscript.

## Supplementary material

Supplementary Figure 1 Mean change from baseline for **(A)** total fatigue, **(B)** cognitive fatigue, and **(C)** motor fatigue as determined by the fatigue scale for motor and cognitive functions (FSMC) at 12- and 24-weeks for the Swank (black bars) and Wahls (gray bars) groups excluding participants who did not adhere to their assigned diet (*n* = 5 Swank, *n* = 8 Wahls) at 12-weeks. Statistical significance was determined by generalized linear mixed models and represented by ^*^*p* ≤ 0.05, ^**^*p* ≤ 0.01, and ^***^*p* ≤ 0.001.

Supplementary Table 1 Metabolic risk factor values among participants with RRMS assigned to the Swank or Wahls dietary interventions excluding participants who were not adherent to their assigned diet.
