# Supplementary figures and images for: Association between improved metabolic risk factors and perceived fatigue during dietary intervention trial in relapsing-remitting multiple sclerosis: A secondary analysis of the WAVES trial
Source: Front Neurol. 2023 Jan 19;13:1022728. doi: 10.3389/fneur.2022.1022728 (PMC9892773; doi:10.3389/fneur.2022.1022728)

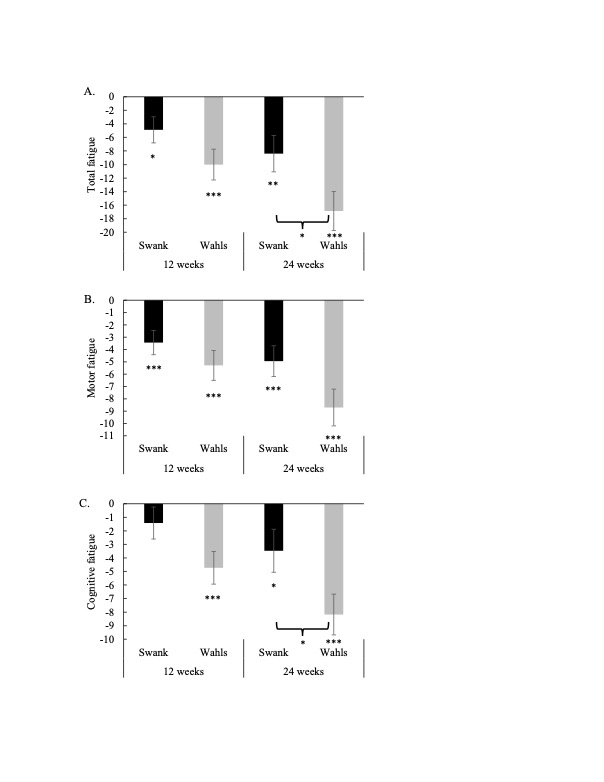

Supplement: Supplementary Figure 1 — Mean change from baseline for (A) total fatigue, (B) cognitive fatigue, and (C) motor fatigue as determined by the fatigue scale for motor and cognitive functions (FSMC) at 12- and 24-weeks for the Swank (black bars) and Wahls (gray bars) groups excluding participants who did not adhere to their assigned diet (n = 5 Swank, n = 8 Wahls) at 12-weeks. Statistical significance was determined by generalized linear mixed models and represented by *p ≤ 0.05, **p ≤ 0.01, and ***p ≤ 0.001. [file Image_1.jpg]
