# Supplementary material for: Association between improved metabolic risk factors and perceived fatigue during dietary intervention trial in relapsing-remitting multiple sclerosis: A secondary analysis of the WAVES trial
Source: Front Neurol. 2023 Jan 19;13:1022728. doi: 10.3389/fneur.2022.1022728 (PMC9892773; doi:10.3389/fneur.2022.1022728)
Supplement: Supplementary Table 1 — Metabolic risk factor values among participants with RRMS assigned to the Swank or Wahls dietary interventions excluding participants who were not adherent to their assigned diet. [file Table_1.docx]

| **Supplemental Table 1.** Metabolic risk factor values among participants with RRMS assigned to the Swank or Wahls dietary interventions excluding participants who were not adherent to their assigned diet. | | | | |
| --- | --- | --- | --- | --- |
|  | **Study visit** | | | |
| Biomarker | Run-in | Baseline | 12 Weeks | 24 Weeks |
| **Swank** |  |  |  |  |
| Systolic BP (mm Hg) | 115 ± 2.54 | 116 ± 2.70 | 114 ± 2.89 | 115 ± 3.31 |
| Diastolic BP (mm Hg) | 75.0 ± 1.77 | 74.1 ± 1.80 | 73.2 ± 1.81 | 72.5 ± 1.99 |
| Weight (kg) | 78.5 ± 3.23 | 78.6 ± 3.24 | 75.4 ± 3.40^***^ | 76.0 ± 3.71^**^ |
| BMI (kg/m^2^) | 28.0 ± 1.02 | 28.0 ± 1.02 | 27.0 ± 1.06^***^ | 26.9 ± 1.16^***^ |
| Glucose (mg/dL) | 92.8 ± 1.39 | 92.9 ± 1.54 | 90.7 ± 1.65 | 92.1 ± 1.48 |
| A1c (%) | 5.34 ± 0.07 | 5.30 ± 0.07 | 5.32 ± 0.07 | 5.23 ± 0.06 |
| Insulin (µIU/mL) | 6.61 ± 0.84 | 6.83 ± 0.94 | 6.25 ± 1.07 | 6.24 ± 1.13 |
| Cholesterol (mg/dL) | 193 ± 6.04 | 191 ± 6.13 | 170 ± 4.44^***^ | 176 ± 5.47^***^ |
| HDL (mg/dL) | 64.9 ± 3.11 | 61.3 ± 2.43 | 56.5 ± 2.16^***,†^ | 59.3 ± 2.44^†^ |
| LDL (mg/dL) | 125 ± 5.41 | 126 ± 5.54 | 109 ± 4.55^***^ | 111 ± 5.11^***^ |
| Triglycerides (mg/dL) | 96.8 ± 7.95 | 97.2 ± 9.68 | 87.7 ± 7.56 | 85.0 ± 6.86 |
|  |  |  |  |  |
| **Wahls** |  |  |  |  |
| Systolic BP (mm Hg) | 117 ± 2.16 | 117 ± 2.91 | 114 ± 2.42 | 117 ± 2.98 |
| Diastolic BP (mm Hg) | 76.7 ± 1.74 | 77.0 ± 2.00 | 72.6 ± 1.81^**^ | 73.3 ± 2.28^*^ |
| Weight (kg) | 85.6 ± 3.92 | 86.0 ± 3.93 | 80.1 ± 3.68^***^ | 76.5 ± 3.30^***^ |
| BMI (kg/m^2^) | 30.3 ± 1.51 | 30.5 ± 1.52 | 28.5 ± 1.47^***^ | 28.2 ± 1.63^***^ |
| Glucose (mg/dL) | 96.9 ± 2.77 | 96.5 ± 2.45 | 94.7 ± 2.37 | 95.5 ± 2.48 |
| A1c (%) | 5.46 ± 0.12 | 5.37 ± 0.07 | 5.28 ± 0.06 | 5.33 ± 0.08 |
| Insulin (µIU/mL) | 7.71 ± 1.32 | 9.95 ± 1.92 | 4.92 ± 0.56^**^ | 5.01 ± 0.75^**^ |
| Cholesterol (mg/dL) | 193 ± 6.78 | 195 ± 7.22 | 180 ± 6.74^***^ | 185 ± 6.88^*^ |
| HDL (mg/dL) | 66.5 ± 3.09 | 66.5 ± 2.90 | 65.7 ± 3.36^†^ | 69.2 ± 3.32^†^ |
| LDL (mg/dL) | 121 ± 5.95 | 122 ± 6.31 | 109 ± 5.67^***^ | 109 ± 6.45^***^ |
| Triglycerides (mg/dL) | 108 ± 8.96 | 107 ± 9.66 | 82.9 ± 8.73^***^ | 75.0 ± 6.50^***^ |
| All values are mean ± SEM.  Within-group statistical significance compared to baseline values indicated by ^*^ for (P ≤ 0.05), ^**^ for (P ≤ 0.01), and ^***^ for (P ≤ 0.001).  Between-group statistically significant differences indicated by ^†^ for (P ≤ 0.05). | | | | |
